# FLOT1 Is a Novel Serum Biomarker of Ovarian Cancer Targeted by N6‐methyladenosine Modification Inhibition

**DOI:** 10.1002/cbin.70015

**Published:** 2025-03-11

**Authors:** Bin Guan, Qi Lu, Junyu Chen, Jingyi Fang, Zhenyu Liu, Wei Li, Lingyun Zhang, Guoxiong Xu

**Affiliations:** ^1^ Research Center for Clinical Medicine, Jinshan Hospital, Fudan University Shanghai China; ^2^ Department of Oncology Shanghai Medical College, Fudan University Shanghai China; ^3^ Department of Obstetrics and Gynecology Jinshan Hospital, Fudan University Shanghai China; ^4^ Shanghai Yizhi Medical Technology Co. Ltd Shanghai China; ^5^ Department of Medical Oncology Shanghai Geriatric Medical Center Shanghai China; ^6^ Department of Medical Oncology Zhongshan Hospital, Fudan University Shanghai China

**Keywords:** Biomarker, CA125, flotillin, mRNA methylation, tumor formation

## Abstract

Ovarian cancer (OC) is a deadly disease and lacks a precise marker for diagnosis worldwide. Our previous work has shown the overexpression of flotillin‐1 (FLOT1) in OC tissue. To improve diagnostic sensitivity and accuracy, we evaluated the serum level of FLOT1 in OC patients and found that the serum concentration of FLOT1 as well as CA125 was significantly increased in patients with OC compared with healthy control (*p* < 0.01) and those with benign tumors (*p* < 0.05). The detection rate (above the upper limit of a cut‐off value) of FLOT1 and CA125 was 77.78% and 72.22%, respectively, in patients with OC, which was increased to 88.89% in combination. The elevation of FLOT1 was confirmed in the serum of nude mice after the implantation of human OC cells. A high level of FLOT1 protein in the serum was positively correlated with the overexpression of FLOT1 protein in OC tissues. Furthermore, the level of m^6^A modification of FLOT1 mRNA was significantly high in OC cells compared with normal ovarian epithelial cells, leading to an increase in FLOT1 mRNA expression. Application of a methylation inhibitor, 3‐deazaadenosine, decreased FLOT1 mRNA expression in OC cells and suppressed tumor formation in a xenograft mouse model. In conclusion, the current study demonstrated that FLOT1 is a novel serum biomarker of OC and can be targeted by m^6^A modification inhibition. These data highlight the potential application of FLOT1 as a diagnostic marker and a therapeutic target for patients with OC.

**Abbreviations**
3‐DAA: 3‐deazaadenosine
AUC: area under the curve
CA125: carbohydrate antigen 125
CCLE: Cancer Cell Line Encyclopedia
CI: confidence interval
FIGO: International Federation of Gynecology and Obstetrics
FLOT1: flotillin‐1
GEO: Gene Expression Omnibus
GO: Gene Ontology
HE4: human epididymis protein 4
HR: hazard ratio
ICGC: International Cancer Genome Consortium
IHC: immunohistochemistry
KEGG: Kyoto Encyclopedia of Genes and Genomes
MeRIP: methylated RNA immunoprecipitation
OC: ovarian cancer
ROC: receiver operating characteristics
RRID: research resource identification
RT‐qPCR: reverse transcription‐quantitative polymerase chain reaction
SD: standard deviation
TCGA: The Cancer Genome Atlas.

## 1 Introduction

The global burden of cancers increased significantly from 1990 to 2021 with which the incidence of ovarian cancer (OC) increased from 159,096 to 298,876 cases (Li et al. 2025). Among gynecologic malignancies, OC is the second cause of cancer death in the United States (Siegel et al. 2025). Although pelvic examination and transvaginal ultrasonography are used as routine diagnostic procedures, most women have failed to detect OC at an early stage because of lacking symptoms at the early stage (Buys. 2011). Low public awareness and vague symptoms often lead to late diagnosis at advanced disease stages (Davenport et al. 2022). Therefore, finding an early detection marker is important, which may provide an opportunity to treat OC patients at an early stage and ultimately reduce their mortality. For the diagnosis of OC, clinical guidelines recommend to use of a serum biomarker carbohydrate antigen 125 (CA125, also termed cancer antigen 125, carcinoma antigen 125, mucin 16, and MUC16). However, it is neither specific nor sensitive enough for accurate diagnosis as indicated by the National Comprehensive Cancer Network (http://www.nccn.org) because serum CA125 levels are influenced by a multitude of common conditions such as endometriosis, uterine fibroids, pelvic inflammatory disease, and benign ovarian tumor (Ghose et al. 2024). On the other hand, some OC patients are without an elevation of serum CA125 (Klotz et al. 2023, Zhang et al. 2021), indicating that false positives and false negatives of the CA125 test exist clinically.

Our previous report demonstrated that flotillin‐1 (FLOT1) was overexpressed in OC cells (Li et al. 2018). FLOT1 is an integral membrane protein localized to the caveolae and is involved in membrane trafficking, signal transduction, and metastasis (McClellan et al. 2024, Wang et al. 2023). The Human Protein Atlas shows that FLOT1 is a secretory protein detectable in the blood by mass spectrometry (https://www.proteinatlas.org/search/FLOT1). However, nothing is known about FLOT1 concentration in the plasma of OC patients. Furthermore, the regulatory mechanism of FLOT1 mRNA expression in OC remains unknown. It has been shown that N6‐methyladenosine (m^6^A) is a widespread posttranscriptional modification that plays a vital role in regulating mRNA stability, translation, alternative splicing, and subcellular localization (Khan et al. 2024, Wang et al. 2015). Aberrations in m^6^A modification disrupt the regulation of the transcriptome and proteome, thereby influencing a variety of biological processes, including carcinogenesis. For example, m^6^A enhances the stability of oncogenic genes such as c‐Myc, AKT, and PD‐L1 (Liu et al. 2023, Ni et al. 2022, Zeng et al. 2024), which facilitate tumor progression and immune evasion. Based on the role of m^6^A in biological processes and our previous work on FLOT1 in OC, we speculate that m^6^A modification could potentially be involved in FLOT1 expression in tumorigenesis. The level of m^6^A modification can generally be suppressed by 3‐deazaadenosine (3‐DAA), an inhibitor of S‐adenosylhomocysteine hydrolase that influences cellular m^6^A methyltransferase activity (Huang et al. 2021). Treatment with 3‐DAA might reduce the m^6^A level of FLOT1 mRNA, affecting tumor formation.

This study aimed to define the serum level of FLOT1 in OC patients and explore the regulatory mechanism of FLOT1 mRNA expression by a general methylation inhibitor in vitro and in vivo. The consequences may provide insights into the clinical application of FLOT1 as a detective biomarker and a therapeutic target for OC.

## 2 Material and Methods

### 2.1 Patients and Pathological Characteristics

Human subjects were obtained from Jinshan Hospital and Zhongshan Hospital, Fudan University. All patients provided informed consent and underwent cytoreductive surgery. The ethical approval was granted by the Ethics Committee of Jinshan Hospitals of Fudan University (approval no. JYLL‐2013‐019‐01) and the Ethics Committee of Zhongshan Hospital of Fudan University (approval no. B2023‐023 R). The histological type, grade, and stage were classified based on the current criteria of the Tumor, Node, and Metastasis (TNM) classification method from the American Joint Committee on Cancer (AJCC) (Olawaiye et al. 2021). The final diagnosis was made by experienced gynecologists and pathologists according to the International Federation of Gynaecological Oncologists (FIGO) system (Prat and Oncology 2015).

### 2.2 Serum Sample Preparation and Enzyme‐Linked Immunosorbent Assay (ELISA)

The blood samples were collected from 18 healthy controls, 9 patients with benign ovarian tumors, and 18 patients with OC at Jinshan Hospital from September 2012 to November 2016, in which the blood samples from patients with OC were taken on the day before the operation (OC‐before) and 2–7 days after the operation without chemotherapy (OC‐after). The serum was prepared using a serum separator tube and was allowed to clot for 60 min at room temperature before centrifugation at 4500 rpm for 5 min. Finally, samples were immediately frozen and stored at −80°C until use. None of the samples were previously thawed. The concentration of serum FLOT1 and CA125 was measured in 96‐well plates using commercial ELISA kits (Shanghai Enzyme‐linked Biotechnology Co. Ltd, Shanghai, China) according to the manufacturer's recommendations. Briefly, mice and human serum samples were diluted 1:10 and 1:5 with the sample dilution buffer, respectively. After adding 50 µl assay buffer into each well, 50 µl standards and diluted serum samples were added into the wells, respectively, and incubated at room temperature for 30 min. After washing away any unbound substances, 50 µl enzyme‐linked polyclonal antibody was added into each well and incubated at room temperature for 30 min. After washing 5 times, 50 µl color‐substrate solution A and solution B were added, subsequently, into each well and incubated at room temperature for 10 min in the dark. After adding 50 µl stop solution into each well, the optical density (OD) in each well was determined within 15 min at 450 nm using a microplate reader (Bio‐Rad Laboratories Inc., Hercules, CA, USA).

### 2.3 Tissue Sample Preparation and Immunohistochemistry (IHC) Staining

Ovarian tissue specimens were collected from 5 women with benign tumors as control subjects and 10 patients with OC at Zhongshan Hospital from August 2023 to October 2023. Tissue specimens were fixed in 4% paraformaldehyde (PFA), embedded in paraffin, and sectioned (4 µm thick). After deparaffinization in xylene and rehydration in a descending alcohol series, 3% hydrogen peroxide in methanol was applied to quench the endogenous peroxide activity. After blocking with 10% normal goat serum (Fuzhou Maixin Biotech. Co. Ltd., Fuzhou, Fujian, China) for 40 min at room temperature, the sections were incubated with a primary rabbit monoclonal anti‐human FLOT1 antibody (1:100 dilution; RRID: AB_11156367, Cat# ab133497, Abcam, Melbourne, Australia) at 4°C overnight, followed by a HRP‐conjugated anti‐rabbit secondary antibody (1:500 dilution; RRID: AB_2811189; Cat# GB23303, Servicebio, Wuhan, China) for 1 h at room temperature or a rabbit monoclonal anti‐human Ki67 antibody (1:100 dilution; RRID: AB_2834152; Cat# AF0198, Affinity Biosciences, OH, United States). After staining with a DAB kit (Cat# DAB‐1031; Fuzhou Maixin Biotech. Co. Ltd), images were captured using a light microscope (BX43; Olympus Corporation).

The IHC results were evaluated by two independent pathologists. The percentage of positively stained tumor cells was scored as 0 (0%), 1 (less than 1%), 2 (1‐10%), 3 (11‐33%), 4 (34‐66%), and 5 (greater than 66%). Additionally, the staining intensity was scored as 0 (no staining), 1 (weak staining), 2 (moderate staining), and 3 (strong staining). Finally, the IHC staining score (ISS) was calculated by summing the percentage scores and the intensity scores. The IHC results were categorized based on the ISS: negative (ISS < 3), weakly positive (ISS 4–6), and strongly positive (ISS 7–8).

### 2.4 Cell Lines and Culture

Non‐tumorigenic ovarian surface epithelial cell line IOSE‑80 was obtained from the OriGene (OriGene Technologies Inc., Rockville, MD, USA) and two OC cell lines, OVCAR‐3 and A2780, were obtained from the American Type Culture Collection (ATCC, Rockville, MD, USA). IOSE‑80 and OVCAR‐3 were cultured with RPMI‐1640 (VivaCell Biosciences Ltd., Shanghai, China) supplemented with 10% and 20% fetal bovine serum (FBS, VivaCell) respectively. A2780 was cultured with DMEM (VivaCell) supplemented with 10% FBS. All cells were maintained in a humidified cell incubator at 37°C with 5% CO_2_. All cell lines were regularly screened to be pathogen‐ and mycoplasma‐free. The authentication of cell lines was evaluated by short tandem repeat (STR) analysis.

### 2.5 Reverse Transcription‐Quantitative Polymerase Chain Reaction (RT‐qPCR)

Total RNA was extracted using the RNA‐Quick Purification Kit (Shanghai Yishan Biotechnology Co. Ltd.; Cat# ES‐RN001). RT was performed using a PrimeScript RT Master Mix (Roche Diagnostics, Basel, Switzerland). PCR was performed using BeyoFast SYBR Green qPCR Mix (2X; High ROX; Beyotime Biotechnology Inc., Shanghai, China). Sequences of PCR primers are ATTGCCCAGGTAAAAATCCAGG (forward) and CCAAAGAGTGCAAATAGTCCTG (reverse) for FLOT1 and TCATCACCATTGGCAATGAG (forward) and CACTGTGTTGGCGTACAGGT (reverse) for β‐actin. After initial denaturation at 95°C for 1 min, the amplification was done by 40 cycles of denaturation at 95°C for 10 s and annealing/extension at 60°C for 30 s. The threshold cycle (Cq) was determined using the 7300 real‐time PCR system (version 1.4, Applied Biosystems; Thermo Fisher Scientific Inc., Waltham, MA, USA). β‐actin was used as an internal control for normalization and 2^−(∆∆Cq)^ was calculated for the expression level of target genes. Experiments were repeated at least three times.

### 2.6 Total M^6^A Methylated RNA Quantification

EpiQuik™ m^6^A RNA Methylation Quantification Kit (Colorimetric) (Cat# A‐P‐9005; EpiGentek, Farmingdale, NY, USA) was utilized to determine the level of total m^6^A RNA methylation in OC cells. Briefly, 200 ng of total RNA was added to each well with the capture antibody followed by the detection antibody and mixed. The m^6^A content was quantified by calorimetric method at the wavelength of 450 nm. The relative quantification was determined by the relative m^6^A RNA methylation status of two group samples. The calculation for the percentage of m^6^A was carried out using the following formula: m^6^A%=[(SampleOD−NCOD)/S]/[(PCOD − NCOD)/P]×100%, where OD stands for optical density; NC stands for negative control; PC stands for positive input control; S stands for the amount of input sample RNA in ng and P stands for the amount of positive input control in ng.

### 2.7 Methylated RNA Immunoprecipitation (MeRIP)‐qPCR Assay

Total RNA was extracted from OVCAR‐3 and A2780 cells using TRIzol (Thermo Fisher Scientific Inc.) when the cell confluence reached 90%. MeRIP was performed according to the protocol of EpiQuikTM CUT& RUN m^6^A RNA Enrichment Kit (Cat# A‐P‐9018; EpiGentek). Briefly, after concentration measurement, 400 μg RNA was chemically fragmented and diluted in 850 μl IP buffer plus 4 μl RNase Inhibitor. Then, 400 ng RNA was used for input and the remaining RNA (400 μl each) was incubated with N6‐methyladenosine antibody or negative immunoglobulin G (lgG) antibody at 4°C for 4 h and subsequently bound with magnetic beads at 4°C for 2 h according to the manufacturer's instructions. After washing with the IP buffer, a 25:25:1 mixture of phenol, chloroform, and isoamyl alcohol (MilliporeSigma, St. Louis, MO, USA) was applied to extract and purify methylated RNA. Finally, the methylated RNAs were amplified by a PCR method using primers of GAGGGCCATCATGGCCCA (forward) and CTTCCCCAAAGAGTGCAAAT (reverse) to detect methylated FLOT1 mRNA levels.

### 2.8 Treatment of 3‐Deazaadenosine

The methylation inhibitor 3‐DAA was purchased from MedChemExpress (Cat# HY‐W013332; MCE, Shanghai, China). OVCAR‐3 and A2780 cells were treated with 50 μM 3‐DAA for 24 h to inhibit methylation followed by RT‐qPCR analysis. The xenograft mouse model was generated as described below. Mice in the control group were intratumorally injected with 100 µl of saline, while mice in the treated group were intratumorally injected with 10 mg/kg of 3‐DAA every other day for 2 weeks after the tumor reached a volume of approximately 80‐100 mm^3^.

### 2.9 Analysis of Data From the Cancer Cell Line Encyclopedia (CCLE)

The expression matrix of FLOT1 mRNA and protein in OC cell lines was obtained from the CCLE data set (https://portals.broadinstitute.org/ccle) (Gonçalves et al. 2022). The analysis was constructed using the “ggplot2” R package (https://ggplot2.tidyverse.org/) with version 4.0.3 software (v3.3.3) (https://www.r-project.org/foundation/). A *p*‐value < 0.05 was considered statistically significant.

### 2.10 Gene Expression Omnibus (GEO) Data Analysis

All microarray data were downloaded from the GEO DataSets (https://www.ncbi.nlm.nih.gov/gds). The raw data were from MINiML files. Box plots were drawn by boxplot. The sample size of each cohort is listed in the figure legend. Screen conditions were as: *p* < 0.05, fold change > 1.5; gene, FLOT1; data type, mRNA; sample type: clinical specimen; cancer type, ovarian cancer; analysis type, ovarian cancer versus normal analysis or ovarian cancer histology analysis. The “boxplot” R package (https://github.com/sgratzl/chartjs-chart-boxplot) was used to draw Box plots. A *p*‐value < 0.05 was considered statistically significant.

### 2.11 Analyzes of the Cancer Genome Atlas (TCGA), Immune Score, and Gene Set Functional Enrichment Analyzes

RNA‐sequencing expression profiles and corresponding clinical information for OC were downloaded from the TCGA data set (https://tcga-data.nci.nih.gov/tcga/) and were analyzed using the “GSVA” R package (https://github.com/rcastelo/GSVA) with parameter as method = ‘ssgsea’. The correlation between genes and pathway scores was analyzed by Spearman correlation. All the analysis methods and R packages were implemented by R version 4.0.3. To evaluate the reliable results of the immune score, the “immuneeconv” R package version 4.0.3 integrating the CIBERSORT algorithm was used (https://rdocumentation.org/packages/immunedeconv/versions/2.0.3). For gene set functional enrichment analysis, the FLOT1‐related gene sets in 376 OC tissues were obtained from the TCGA portal, compared to a total of 180 normal ovary tissues from the GTEx database (https://www.gtexportal.org/). FLOT1 mRNAs with fold change ≥ 2 and a *p*‐value < 0.05 were considered to be differentially expressed. The FLOT1‐related molecular functions and signaling pathways were evaluated by Gene Ontology (GO) and Kyoto Encyclopedia of Genes and Genomes (KEGG) analyzes. Furthermore, a Pearson method determined the correlation between FLOT1 and m^6^A regulators in the datasets. The square of correlation coefficient |R2 | > 0.5 and *p*‐value < 0.05 were used to identify FLOT1‐related m^6^A regulators.

### 2.12 International Cancer Genome Consortium (ICGC) Data Analysis

RNA‐sequencing expression profiles and corresponding clinical information for OC were downloaded from the ICGC data set (https://platform.icgc-argo.org/). The Log‐rank test was used to compare differences in survival between these groups and was analyzed using the “ggrisk”, “survival”, and “survminer” R packages (https://cran.r-project.org/web/packages/survminer/index.html). The time‐dependent Receiver Operating Characteristic curve analysis was used to compare the predictive accuracy of each gene. For Kaplan‐Meier curves, P‐values and hazard ratio (HR) with 95% confidence interval (CI) were measured by Log‐rank tests and univariate Cox proportional hazards regression using the “timeROC” R package (https://cran.r-project.org/web/packages/timeROC/index.html).

### 2.13 Xenograft Mouse Model

Approval for the animal study was granted by the Ethics Committee of Shanghai Public Health Clinical Center, Fudan University (approval no. GWLL2020‐A0270‐01). For animal injection, 5×10^6^ OVCAR‐3 cells were suspended in 100 µl RPMI‐1640 medium without FBS. A total of 30 female BALB/c nude mice (5‑ to 6‑week‑old, weight 17–20 g; Shanghai Super‑B&K Laboratory Animal Corp. Ltd. Shanghai, China) received water and food ad libitum and were provided constant temperature (22°C–25°C), humidity (50–60%), and a 12‑h light/dark cycle in the animal facility. Evaluation of the serum FLOT1 concentration in nude mice with implantation of OC cells: mice were randomly divided into two groups (*n* = 10 per group): mice without cell injection (Blank group), and mice subcutaneously injected into the right flank of mice with OVCAR‐3 cells (OVCAR‐3 group). Tumor‐bearing mice lived for 21 days. Tumor initiation and progression as well as mouse weight were monitored every other day. Tumor volume was calculated by the formula V=ab^2^/2, where a and b are two dimensions in length and width, respectively. At the end of the experiment, animals were killed and the tumors were excised. The blood was obtained via cardiac puncture and was collected into EDTA‐containing tubes. Mouse serum samples were allowed to clot for 60 min at room temperature and then centrifuged at 4500 rpm for 5 min. Finally, samples were aliquoted and stored at −80°C until use.

To evaluate the effect of a methylation inhibitor 3‐DAA on tumor formation, 5 × 10^6^ OVCAR‐3 cells in 100 µl medium were subcutaneously injected into the right flank of nude mice (*n* = 5/group). Two weeks later, mice were treated with either vehicle or 3‐DAA (10 mg/kg) by intra‐tumoral injection every 2 days for 6 times. Tumor initiation and tumor progression were monitored every other day for 12 days. Tumor volume was calculated and mice were killed after anesthesia with an overdose of sodium pentobarbital intraperitoneal injection. Animals were photographed. The tumors were excised and images were captured.

### 2.14 Protein Extraction and Western blot Analysis

Total proteins were extracted using an SDS Lysis Buffer (Beyotime) with 1% phenylmethanesulfonyl fluoride and 1% phosphatase inhibitor (KeyGen Biotech Co. Ltd., Nanjing, Jiangsu, China). The proteins were separated on 10% SDS‐PAGE, transferred to a PVDF membrane, and incubated with a primary antibody overnight. Primary antibodies used included rabbit anti‐FLOT1 (1:10,000 dilution; RRID: AB_11156367, Cat# ab133497, Abcam, Melbourne, Australia) and mouse anti‐GAPDH (1:10,000 dilution; RRID: AB_2107436, Cat. 60004‐1, Proteintech Group Inc, Wuhan, China). Secondary antibodies used included horseradish peroxidase‐linked goat anti‐rabbit IgG and anti‐mouse IgG (1:5000 dilution; Proteintech). The signals were visualized with BeyoECL Moon from Beyotime (Cat#P0018FS; Beyotime) and quantified with ImageJ software.

### 2.15 Statistical Analysis

Statistical analyzes were performed with SPSS Statistics 21.0 (SPSS, Chicago, IL, USA). For comparison between the two groups, a Student *t*‐test was applied. For comparison of FLOT1 and CA125 expression associated with the clinicopathological characteristics of patients with OC, Fisher's exact test was applied. Receiver operating characteristics (ROC) analysis was performed and ROC curves were used to evaluate the sensitivity, specificity, area under the ROC curve (AUC), and its corresponding 95% confidence intervals (CI) between OC and healthy control groups in humans and mice. Youden's index (YI) was calculated by determining cut‐off values from upper or lower 95% CI of mean in OC compared to healthy controls. Sensitivity and specificity were calculated based on the number of cases that meet the conditions of lower or higher than the cut‐off values. For testing the correlation between different serum markers, a linear regression was applied. The Wilcoxon test was used to assess the immune score between two‐group comparisons. To validate a predictive potential, univariate and multivariate analyzes were performed. Results are presented as the mean ± standard deviation (SD). The significant difference was considered at the value of *p* < 0.05.

## 3 Results

### 3.1 Elevation of Serum FLOT1 and CA125 Levels in Patients With Ovarian Cancer

**Figure 1:**
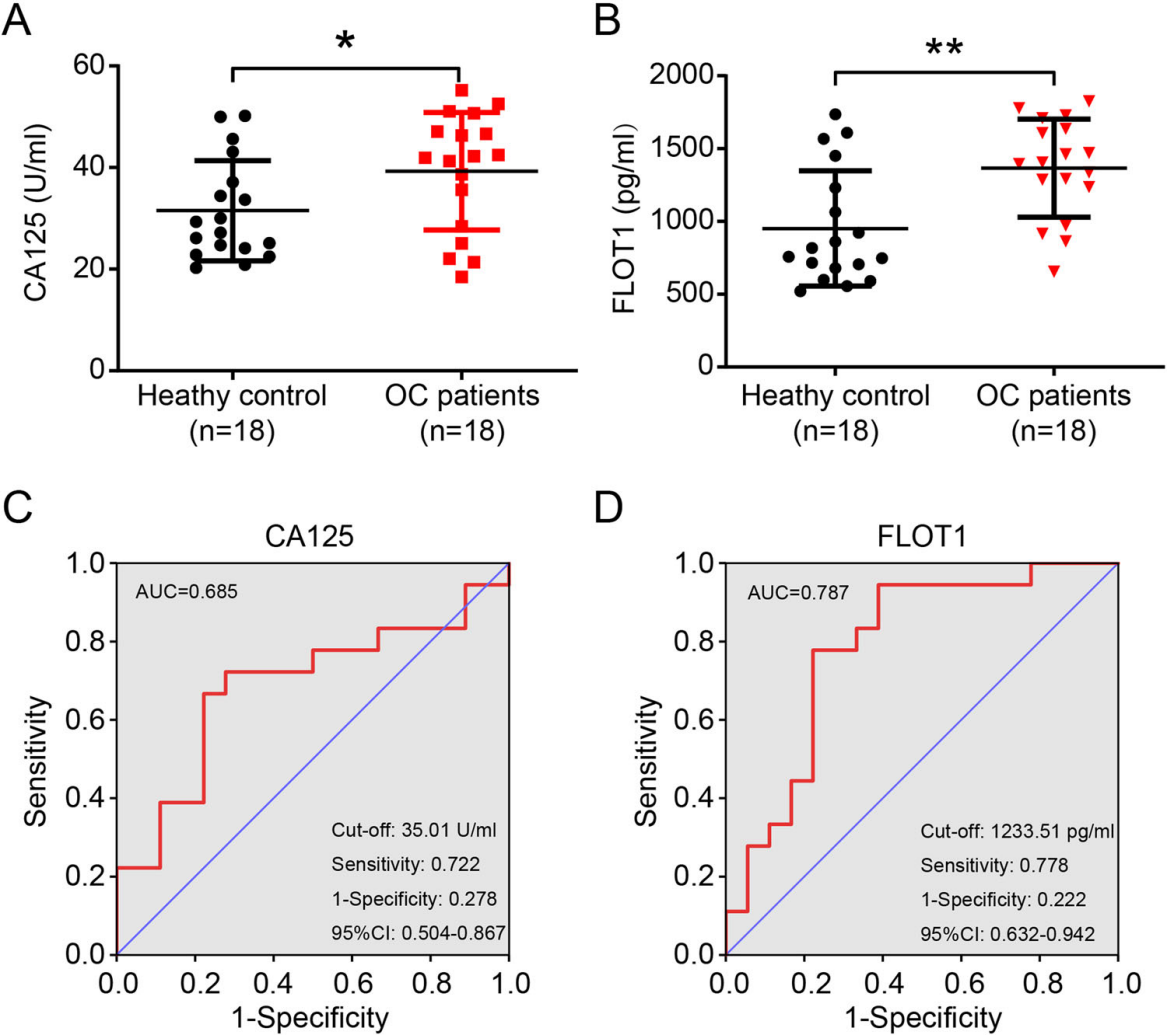
Evaluation of serum CA125 and FLOT1 in humans. (A) Concentration of serum CA125 in healthy controls and OC patients. (B) Concentration of serum FLOT1 in healthy controls and OC patients. (C) ROC analyzes of CA125 in controls versus patients with OC. (D) ROC analyzes for serum FLOT1 in controls versus patients with OC. Area under the ROC curve (AUC), cut‐off, sensitivity, 1‐specificity, and 95% confidence interval (CI) were calculated. Data are presented as the mean ± SD. OC, ovarian cancer; *p < 0.05; **p < 0.01.

The concentrations of serum FLOT1 and CA125 were measured in 18 healthy controls and 18 patients with OC by ELISA. The median age of patients was about 50 years matched to the controls and no significant difference in age was observed between healthy controls and patients. The level of serum CA125, known as an OC biomarker, was elevated in patients with OC compared with healthy controls (*p* < 0.05; Figure 1A). Interestingly, serum FLOT1 was also significantly higher in patients with OC than in healthy controls (*p* < 0.01; Figure 1B). To determine the overall accuracy of the test (sensitivity and specificity), we calculated Youden's index (YI) by determining the cut‐off value in patients with OC compared to healthy controls. The YI of CA125 and FLOT1 was 0.444 and 0.556 in OC, respectively. The cut‐off value of CA125 was 35.01 U/ml, which was similar to its upper limit of normal value (35 UI/ml) set clinically (Janas 2021). An optimal cut‐off value of FLOT1 was 1233.51 pg/ml, which was of critical importance to accurate the diagnosis of OC determined by the score closest to the value of sensitivity (0.778) and 1‐specificity (0.222). Next, we calculated the ROC by plotting the sensitivity against the specificity in patients with OC. The outcomes between CA125 and FLOT1 were similar (Figure 1C,D), indicating that serum FLOT1 as well as serum CA125 is effective to be a diagnostic marker of OC. Further, we validated the predictive potential of FLOT1. Univariate and multivariate analyzes using logistic regression models did not estimate the relationship of FLOT1 independently on the clinical features (Table 1).

**Table 1:** Association of serum FLOT1 protein with the clinical features of patients with ovarian cancer.

Clinical features	Univariate		*p* value	Multivariate		*p* value
	OR	95% CI		OR	95% CI	
Age	1.036	0.943 ~ 1.162	0.4851	1.039	0.936 ~ 1.175	0.4812
Stage	1.386	0.510 ~ 4.847	0.5441	0.609	0.086 ~ 2.751	0.5325
Pathological type	0.323	0.066 ~ 1.253	0.1111	0.287	0.018 ~ 1.921	0.2499
CA125 levels	1.060	0.960 ~ 1.185	0.2594	1.024	0.889 ~ 1.185	0.7223

*Note:* Univariate and multivariate logistic regression analyzes were used to compare clinical characteristics with *p* < 0.05 as the threshold of significance.

Abbreviations: CI, confidence interval; OR, odds ratio.

**Figure 2:**
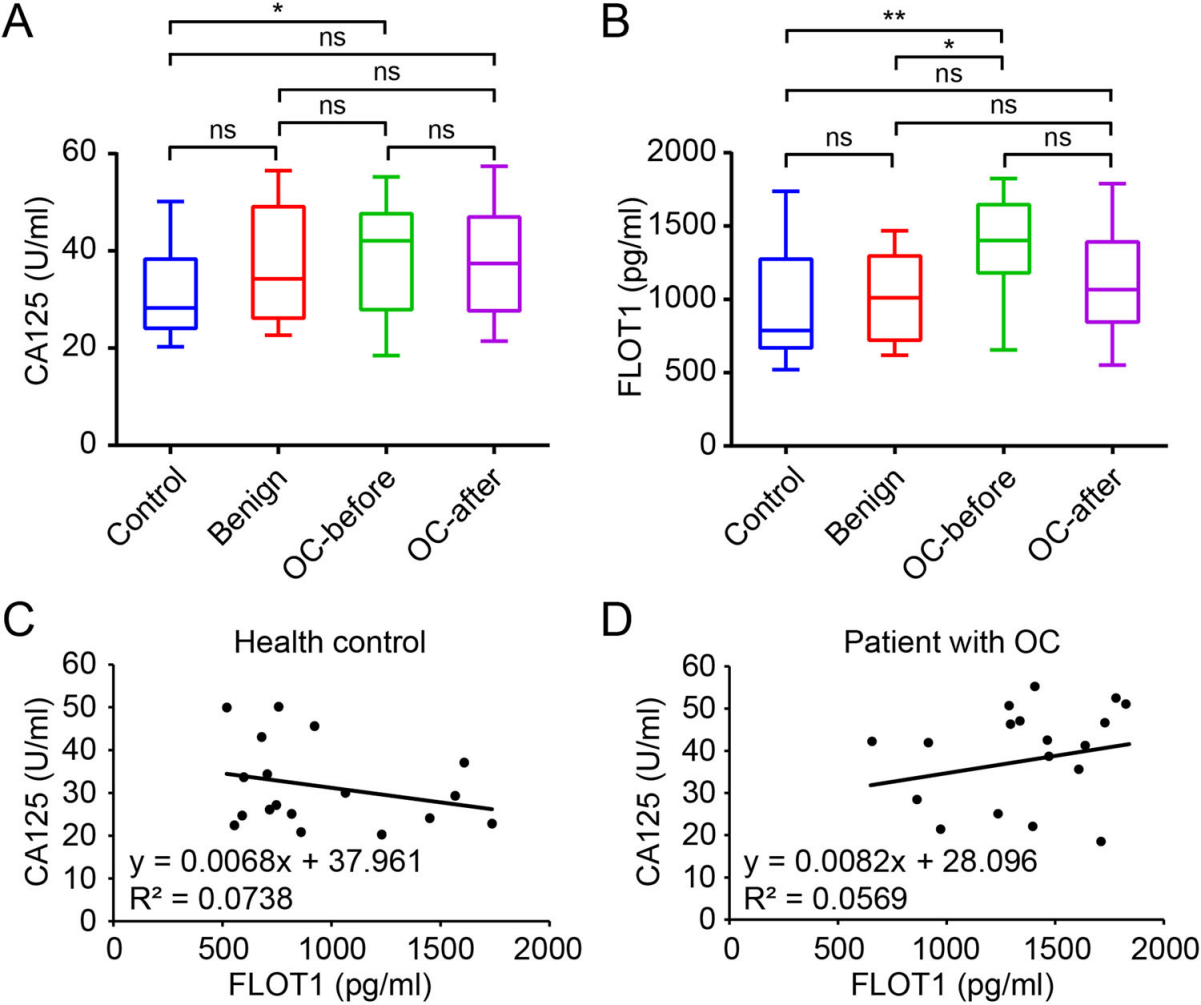
Comparison and correlation of serum CA125 and FLOT1 in human serum. (A) CA125 was detected in healthy controls (Control; n = 18), patients with benign tumors (Benign; n = 9), patients with OC (n = 18) before surgery (OC‐before), and patients with OC after surgery (OC‐after) by ELISA. (B) FLOT1 was detected in Control, Benign, OC‐before, and OC‐after by ELISA. The correlation between the variables was examined by a linear regression. (C) CA125 versus FLOT1 in healthy controls. (D) CA125 versus FLOT1 in patients with ovarian cancer. Data are presented as mean ± SD. ns, no significance; *p < 0.05; **p < 0.01.

Next, we measured and compared the serum levels of FLOT1 and CA125 between healthy controls, patients with ovarian benign tumors, and patients with OC before and after the operation. The concentration of CA125 was significantly higher in patients with OC before the operation compared to healthy control (*p* < 0.05; Figure 2A). Similarly, a high concentration of FLOT1 was also observed in patients with OC before the operation (Figure 2B). Serum FLOT1 was significantly increased in patients with OC compared with healthy control (*p* < 0.01) and patients with benign tumors (*p* < 0.05). After the operation, serum FLOT1 tended to be decreased as there was no significant difference compared to health control. Next, we compared the CA125 variable to the FLOT1 variable to see whether the increase of CA125 and FLOT1 in OC is correlated. However, no significant correlation between CA125 and FLOT1 was observed in healthy controls (*p* = 0.275; Figure 2C) and patients with ovarian OC (*p* = 0.326; Figure 2D).

Subsequently, the detection rate of CA125 and FLOT1 was evaluated using the receiver operating characteristic (ROC) curve analysis. We found that the positive rate in OC detection was increased in combination with FLOT1 and CA125. The detection rate (above the upper limit of a cut‐off value) of CA125 and FLOT1 was 72.22% (*n* = 13/18) and 77.78% (*n* = 14/18), respectively, in patients with OC (Table 2), which was increased to 88.89% (*n* = 16/18) in combination. At the early stages of OC (FIGO I‐II), the positive rate of CA125 and FLOT1 was 70.00% (*n* = 7/10 each), which was increased up to 90.00% (n = 9/10) when the two variables were assessed together. At the late stages of OC (FIGO III–IV), the positive rate of CA125 and FLOT1 was 75.00% (*n* = 6/8) and 87.50% (*n* = 7/8), respectively. These data showed that a panel of FLOT1 and CA125 is highly valuable for the diagnosis of OC.

**Table 2:** Correlation between CA125 and FLOT1 in patients with ovarian cancer.

	FLOT1 (high) *n* (%)	FLOT1 (low) *n* (%)	Total	*p* value
All patients				0.533
CA125 (high)	11 (61.11)	2 (11.11)	13 (72.22)	
CA125 (low)	3 (16.67)	2 (11.11)	5 (27.78)	
Total	14 (77.78)	4 (22.22)	18 (100.00)	
FIGO stage I–II				1.000
CA125 (high)	5 (50.00)	2 (20.00)	7 (70.00)	
CA125 (low)	2 (20.00)	1 (10.00)	3 (30.00)	
Total	7 (70.00)	3 (30.00)	10 (100.00)	
FIGO stage III–IV				0.250
CA125 (high)	6 (75.00)	0 (0.00)	6 (75.00)	
CA125 (low)	1 (12.50)	1 (12.50)	2 (25.00)	
Total	7 (87.50)	1 (12.50)	8 (100.00)	

*Note:* For a comparison of FLOT1 to CA125, a Fisher's exact test was applied. High, above the upper limit of a cut‐off value of CA125 (35.01 U/ml) and FLOT1 (1233.51 pg/ml); low, under the upper limit of a cut‐off value of CA125 and FLOT1; *n*, number of cases.

### 3.2 Confirmation of FLOT1 Elevation in the Serum of Nude Mice after Implantation of Ovarian Cancer Cells

**Figure 3:**
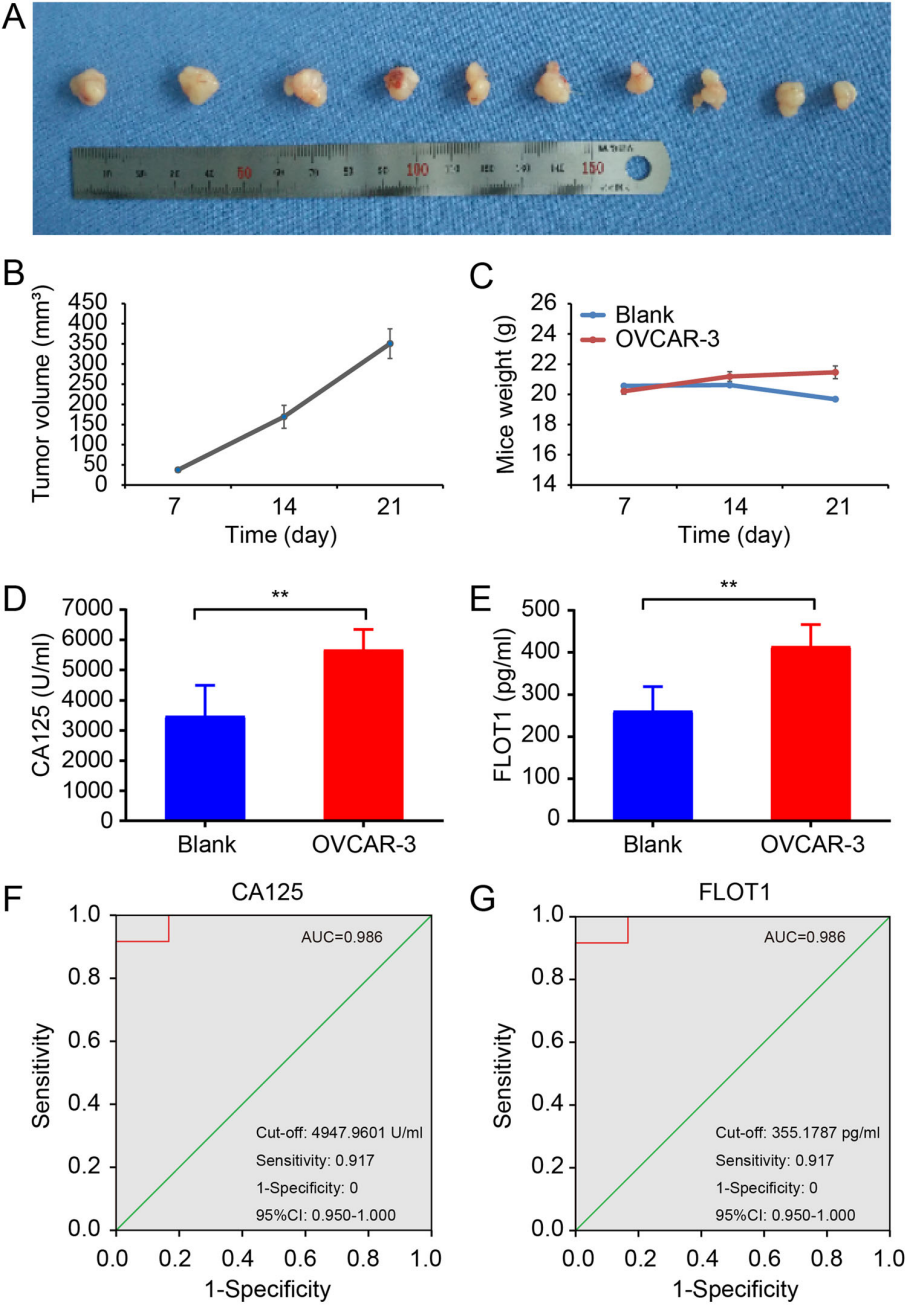
Evaluation of FLOT1 concentration and ROC analyzes of the sensitivity against the specificity for CA125 and FLOT1 proteins in nude mice. Mice were implanted with OVCAR‐3 cells subcutaneously and lived for 21 days. (A) Photograph of excised tumor mass after sacrifice. (B) Measurement of tumor volume in a time‐course study. The average tumor volume in OVCAR‐3 implanted mice was about 350 mm^3^. (C) Measurement of mouse weight in a time‐course study. The weight of OVCAR‐3 implanted mice (OVCAR‐3) was significantly increased compared to blank control mice (Blank). (D) Measurement of serum CA125 concentration in Blank and OVCAR‐3 mice by ELISA. (E) Measurement of serum FLOT1 concentration in Blank and OVCAR‐3 mice by ELISA. Data are presented as the mean ± SD (*n* = 10). **p < 0.01. (F) Analysis of the area under the ROC curve (AUC), sensitivity, and 1‐specificity of CA125 in OVCAR‐3 implanted mice. (G) Analysis of the AUC, sensitivity, and 1‐specificity of FLOT1 in OVCAR‐3 implanted mice.

Using an animal xenograft model, we measured the serum levels of FLOT1 as well as CA125. Nude mice were injected with OVCAR‐3 cells subcutaneously (*n* = 10). The tumor‐bearing animals were killed on day 21 after the cell transplant. The xenograft tumors were removed and photographed (Figure 3A). The average tumor volume in OVCAR‐3 subcutaneously injected mice was about 350 mm^3^ (Figure 3B). Next, the mouse weight was measured in a time‐course study. We found that the weight of OVCAR‐3 subcutaneously injection mice was significantly increased compared with blank control mice (*p* < 0.05; Figure 3C). These data indicated that the animal xenograft models were successfully generated. ELISA showed that FLOT1 and CA125 were significantly elevated in the OVCAR‐3 implanted mice compared with blank mice (*p* < 0.01; Figure 3D,E). To evaluate serum FLOT1 proteins as OC diagnostic biomarkers, we calculated the ROC curve by plotting sensitivity against specificity in nude mice. The AUC area yielded by the ROC curve of FLOT1 and CA125 with 95% CI was generated in OVCAR‐3 implanted mice and was the same between FLOT1 and CA125 (both AUC = 0.986) (Figure 3F,G).

### 3.3 High Expression of FLOT1 in Human Ovarian Cancer Tissues

**Figure 4:**
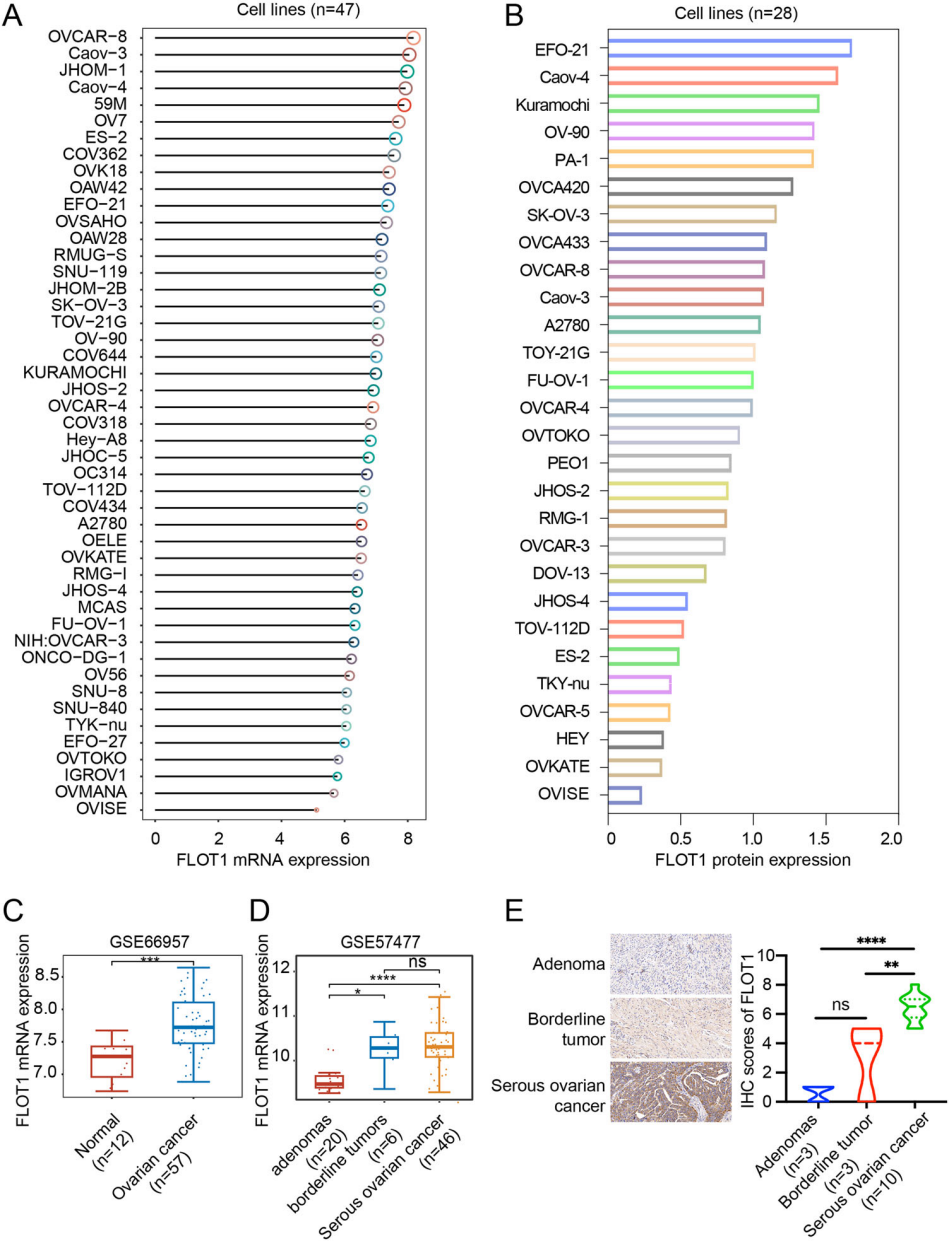
FLOT1 expression in human ovarian cancer. (A) Bar chart of FLOT1 mRNA expression in 47 different ovarian cancer cell lines from CCLE (https://portals.broadinstitute.org/ccle). The abscissa represents different cell lines and the ordinate represents the level of FLOT1 mRNA expression. (B) Bar chart of FLOT1 protein expression in 28 different ovarian cancer cell lines by mass spectrometry. (C) FLOT1 mRNA expression in ovarian cancer and ovarian surface epithelium tissue (Normal) from a GEO data set (GSE66957; http://www.ncbi.nih.gov/geo). The Wilcoxon test was used for comparison. ***p < 0.001. (D) FLOT1 mRNA expression in ovarian adenomas, borderline tumors, and serous ovarian adenocarcinomas from a GEO data set (GSE57477). The Kruskal‐Wallis test was used for multi‐group comparison. ns, not significance; *p < 0.05; ****p < 0.0001. (E) Immunohistochemical staining of FLOT1 protein in ovarian adenomas, borderline tumors, and serous ovarian adenocarcinoma tissues from hospitalized patients after surgery.

Through the analysis of the CCLE database, we found that FLOT1 mRNA was highly overexpressed in 47 human OC cell lines (Figure 4A). Further analysis of protein expression showed that FLOT1 was highly expressed in most of the 28 OC cell lines (Figure 4B). Transcriptome analysis of the online GEO data set (GSE66957) showed that the level of FLOT1 mRNA expression was higher in OC tissues (*n* = 57) than in ovarian surface epithelium tissue (Normal, *n* = 17) (Figure 4C). Compared to the different ovarian tumors from another GEO data set (GSE57477), we found that the levels of FLOT1 mRNA expression were higher in serous ovarian adenocarcinomas (*n* = 46) and borderline tumors (*n* = 6) than in benign adenomas (*n* = 20) (Figure 4D). IHC confirmed the overexpression of FLOT1 protein in OC tissue as shown strong staining in serous ovarian adenocarcinomas was observed (Figure 4E), which was consistent with the data from the GEO database.

### 3.4 Association of Serum FLOT1 Protein With the Clinicopathological Features of Patients With Ovarian Cancer

**Figure 5:**
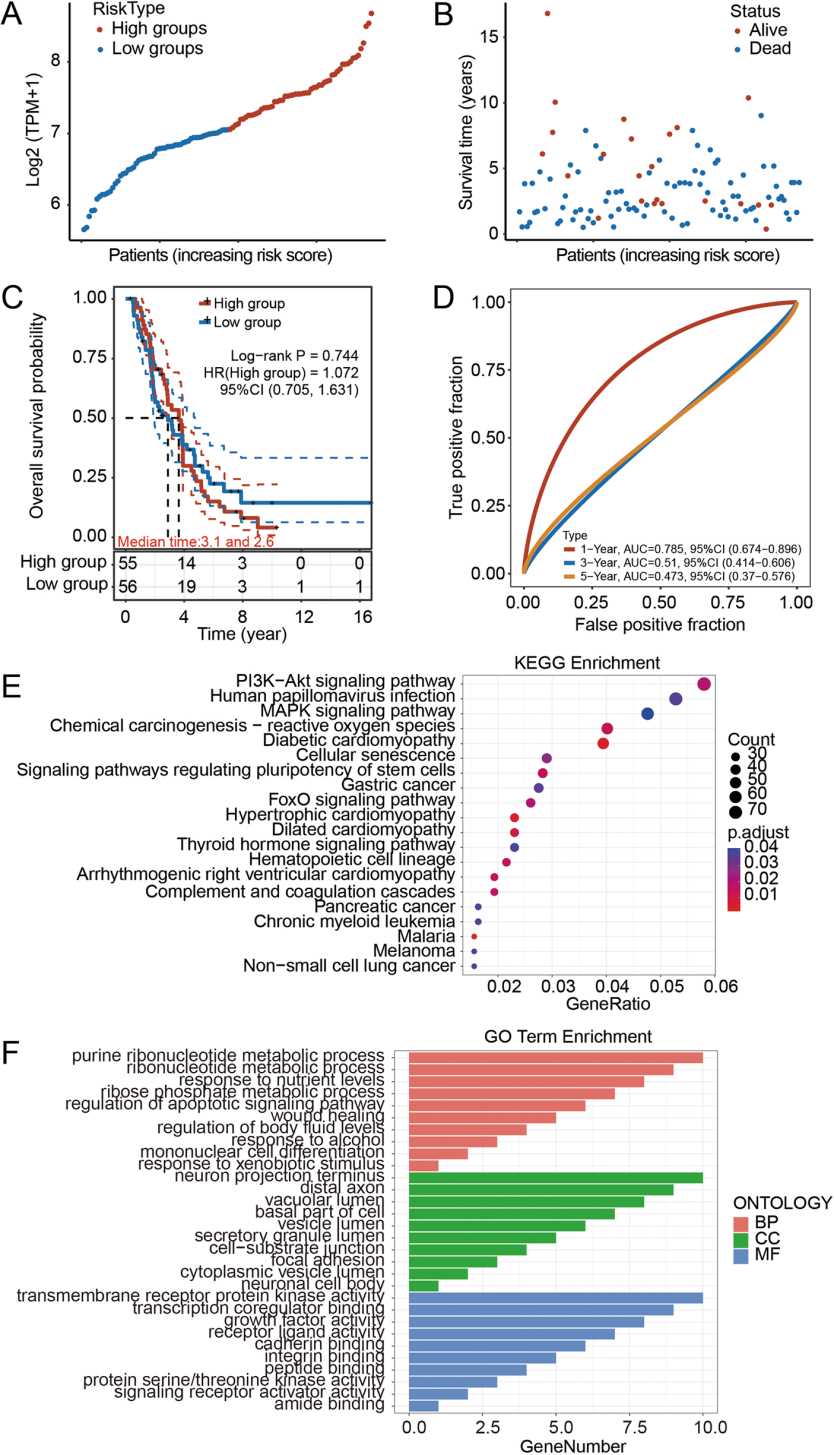
FLOT1 expression with OC survival rate. (A) The gene expression, survival time, and survival status of the ICGC data set (https://platform.icgc-argo.org/). Patients were distinguished by the median value of FLOT1 expression and the scatterplot represents the gene expression from low to high. Different colors represent different groups. (B) FLOT1 expression, survival time, and survival status of the ICGC data set. The scatter plot distribution represents the gene expression of different samples corresponding to the survival time and survival status. (C) Kaplan‐Meier survival analysis of the gene signature from the ICGC data set, comparison among different groups was made by log‐rank test. HR (High exp) represents the hazard ratio of the low‐expression sample relatives to the high‐expression sample. HR > 1 indicates the gene is a risk factor, whereas HR < 1 indicates the gene is a protective factor. HR (95%Cl), the median survival time for different groups. (D) The ROC curve of the gene. The higher values of AUC corresponded to higher predictive power. (E) KEGG analyzes of FLOT1‐correlated genes function enrichment. (F) GO analyzes of FLOT1‐correlated genes function enrichment. BP, biological process; CC, cellular component; MF, molecular function.

The median age of patients with ovarian tumors and healthy controls was 49.8 years (range: 27–73 years) and 51.8 years (range: 43–63 years), respectively. The age of patients was matched to healthy controls for comparison (*p* = 0.691987). The clinical information was found by reviewing medical charts and pathology records. The association of serum FLOT1 and CA125 with the clinicopathological features of patients with OC was further examined. Multiple comparisons showed that serum FLOT1 and CA125 levels were not associated with ages, histological types, grades, and clinical stages in patients with OC (*p* > 0.05; Table 3). Because the sample size of patients with malignant tumors was relatively small (a total of 18 cases), an increase in the sample number must be considered in a future study. From the ICGC database, we found that the distribution of the corresponding scatter plots from left to right showed no significant difference when the FLOT1 expression was ranked from low to high, (Figure 5A,B). OC patients with overexpressed FLOT1 had no significant change of OS values to low expressed (Figure 5C). In terms of time‐dependent ROC curves, 1, 3, and 5 years of survival can be assessed using Area Under Curve (AUC) values that were 0.785, 0.51, and 0.473, respectively. The high expression group of FLOT1 was closely linked with a worse 1‐year OS of OC patients compared to 3‐ and 5‐year OS of OC patients (Figure 5D), indicating that the predictive power of FLOT1 varied with patient survival time. The differential expression genes (DEG) correlated to FLOT1 were analyzed using the KEGG pathway database. The enrichment analysis revealed that among the top 20 pathways, the Pl3K‐Akt signaling pathway was the most significant pathway with a rich factor of 0.058, while the maximum number of the DEGs were mainly related to the Pl3K‐Akt signaling, MAPK signaling, and FoxO signaling pathways, etc, and were involved in viral infection, cardiovascular disease, and cancer (Figure 5E). The GO term analysis showed that the DEGs are mainly distributed in the neuron projection terminus, distal axon, vacuolar lumen, basal part of cells, vesicle lumen, and secretory granule lumen (Figure 5F). They participate in regulating purine ribonucleotide metabolic process, ribonucleotide metabolic process, response to nutrient levels, ribose phosphate metabolic process, regulation of apoptotic signaling pathway, wound healing, regulation of body fluid levels, response to alcohol, mononuclear cell differentiation, response to the xenobiotic stimulus. Their functions were mainly involved in transmembrane receptor protein kinase activity, transcription coregulator binding, growth factor activity, receptor‐ligand activity, cadherin binding, integrin binding, peptide binding, protein serine/threonine kinase activity, signaling receptor activator, amide binding. These data indicate that FLOT1 is involved in ribonucleotide metabolism, apoptosis, and receptor/ligand activity, potentially regulating the tumorigenesis of OC.

**Table 3:** Association of serum FLOT1 protein with the clinicopathological features of patients with ovarian cancer.

Clinicopathological features	*n*	FLOT1 detection			CA125 detection		
		High	Low		High	Low	
		*n* (%)	*n* (%)	*p* value	*n* (%)	*n* (%)	*p* value
Age at diagnosis				0.577			1.000
≤ 45	9	6 (66.67)	3 (33.33)		7 (77.78)	2 (22.22)	
> 45	9	8 (88.89)	1 (11.11)		6 (66.67)	3 (33.33)	
Histological type				0.533			1.000
Serous carcinoma	13	11 (84.62)	2 (15.38)		10 (76.92)	3 (23.08)	
Others*	5	3 (60.00)	2 (40.00)		4 (80.00)	1 (20.00)	
Grade (serous)				1.000			0.423
High	11	9 (81.82)	2 (18.18)		9 (81.82)	2 (18.18)	
Low	2	2 (100.00)	0 (0.00)		1 (50.00)	1 (50.00)	
FIGO stage				0.588			1.000
I–II	10	7 (66.67)	3 (33.33)		7 (70.00)	3 (30.00)	
III–IV	8	7 (87.50)	1 (12.50)		6 (75.00)	2 (25.00)	

*Note:* The serum FLOT1 and CA125 proteins were detected by ELISA. For comparison of FLOT1 and CA125 proteins in serum associated with age, histological type, grade (serous carcinoma), and clinical stage, a Fisher's exact test was applied. *, other histological types of OC, including 3 cases of clear cell ovarian cancer (OC), 1 case of germ cell OC, and 1 case of metastatic OC. High, above the upper limit of a cut‐off value of CA125 (35.01 U/ml) and FLOT1 (1233.51 pg/ml); low, under the upper limit of a cut‐off value of CA125 (normal) and FLOT1; n, number of cases; FIGO, International Federation of Gynaecological Oncologists.

**Figure 6:**
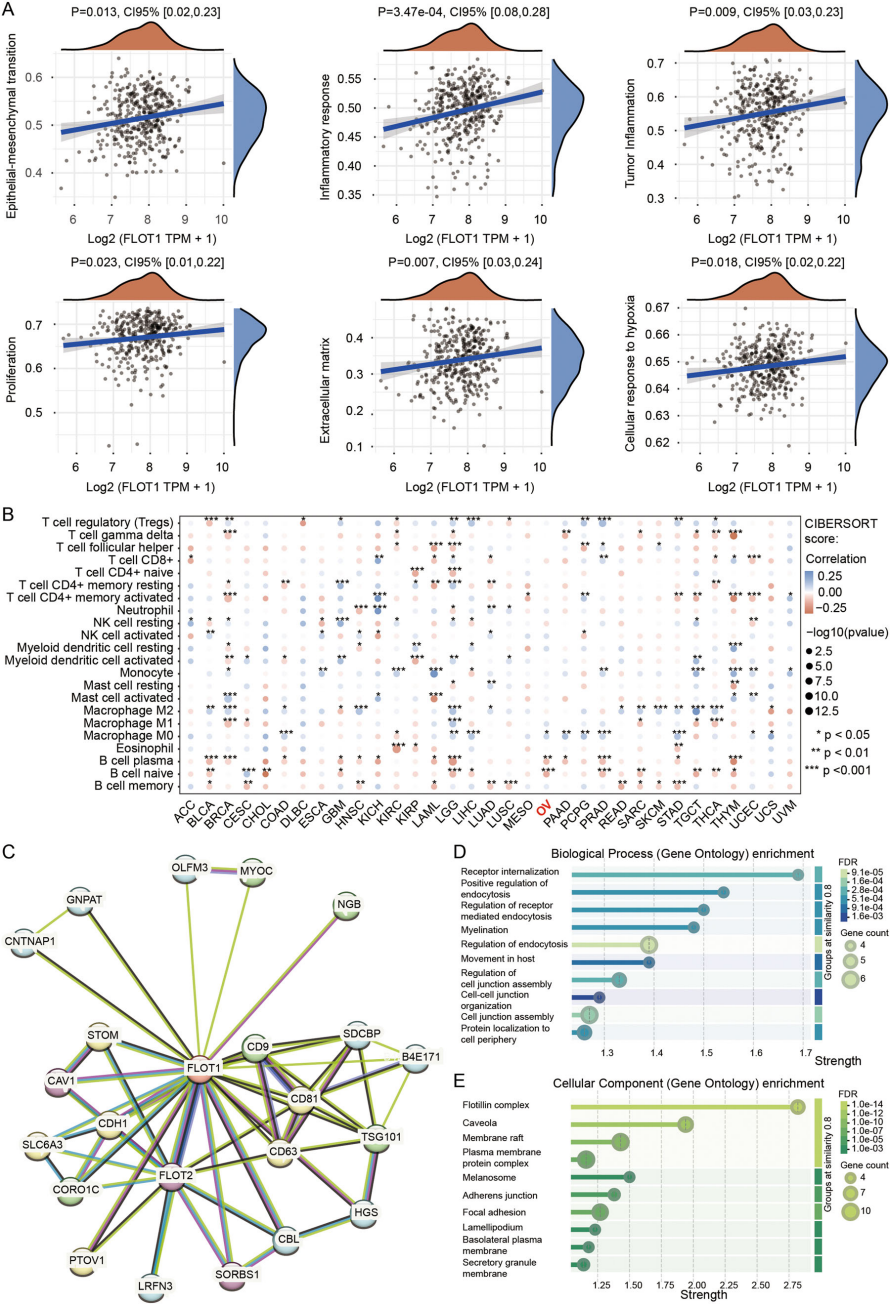
Evaluation of the FLOT1 function in the data from the TCGA database. (A) The correlations between individual genes and pathway scores were analyzed with Spearman. The abscissa represents the distribution of the gene expression and the ordinate represents the distribution of the pathway score. The density curve on the right represents the trend in the distribution of the pathway score. The upper‐density curve represents the trend in the distribution of the gene expression. The value on the top represents the correlation *P*‐value and 95%CI. The function of FLOT1 was mainly related to epithelial‐mesenchymal transition (EMT), inflammatory response, tumor inflammation, proliferation, extracellular matrix (ECM) related genes, and cellular response to hypoxia. (B) Measurement of immune correlations. The heatmap of the immune score and FLOT1 expression in pan‐cancers. The abscissa represents different tumor tissues, and the ordinate represents different immune scores detected by the CIBERSORT algorithm. Different colors represent the correlation coefficients. Negative values indicate negative correlations and positive values indicate positive correlations. The deeper color represents the stronger correlation. Asterisks (*) stand for significance levels. The statistical difference between the high and low groups was compared through the Wilcoxon test. (C) The protein‐protein interaction (PPI) network revealed 23 nodes and 57 edges (P = 2.53e‐09) The differential expression of FLOT1 was significantly associated with FLOT2, SORBS1, and CAV1 with predicted functional scores of 0.993, 0.986, and 0.972, respectively. (D) The GO term analysis revealed that FLOT1 was mainly involved in receptor internalization (Biological Process: *p* = 0.00091). (E) The GO term analysis revealed that FLOT1 was mainly involved in the flotillin complex (Cellular Component: P = 2.48e‐14).

Next, we investigated the biological functions of FLOT1 through the TCGA database. We found that the function of FLOT1 was mainly related to epithelial‐mesenchymal transition (EMT), inflammatory response, tumor inflammation, proliferation, extracellular matrix (ECM) related genes, and cellular response to hypoxia (Figure 6A). In OC, FLOT1 was mainly associated with naïve B cells, plasma B cells, and M0 Macrophages, suggesting that these cells were more infiltrated in patients with high expression with FLOT1 (Figure 6B). The protein‐protein interaction (PPI) network revealed 23 nodes and 57 edges (PPI enrichment P = 2.53e‐09), suggesting a significant functional connection between the identified proteins. The differential expression of FLOT1 was significantly associated with FLOT2, SORBS1, and CAV1 with predicted functional scores of 0.993, 0.986, and 0.972, respectively (Figure 6C). The GO term analysis revealed that FLOT1 was mainly involved in receptor internalization (Biological Process: *p* = 0.00091; Figure 6D) and flotillin complex (Cellular Component: P = 2.48e‐14; Figure 6E). These data indicate that FLOT1 may play a role in the cell membrane involved in endocytosis and cell junction.

### 3.5 Influence of M^6^A Modification on FLOT1 Mrna Expression and Tumor Formation by a Methylation Inhibitor

**Figure 7:**
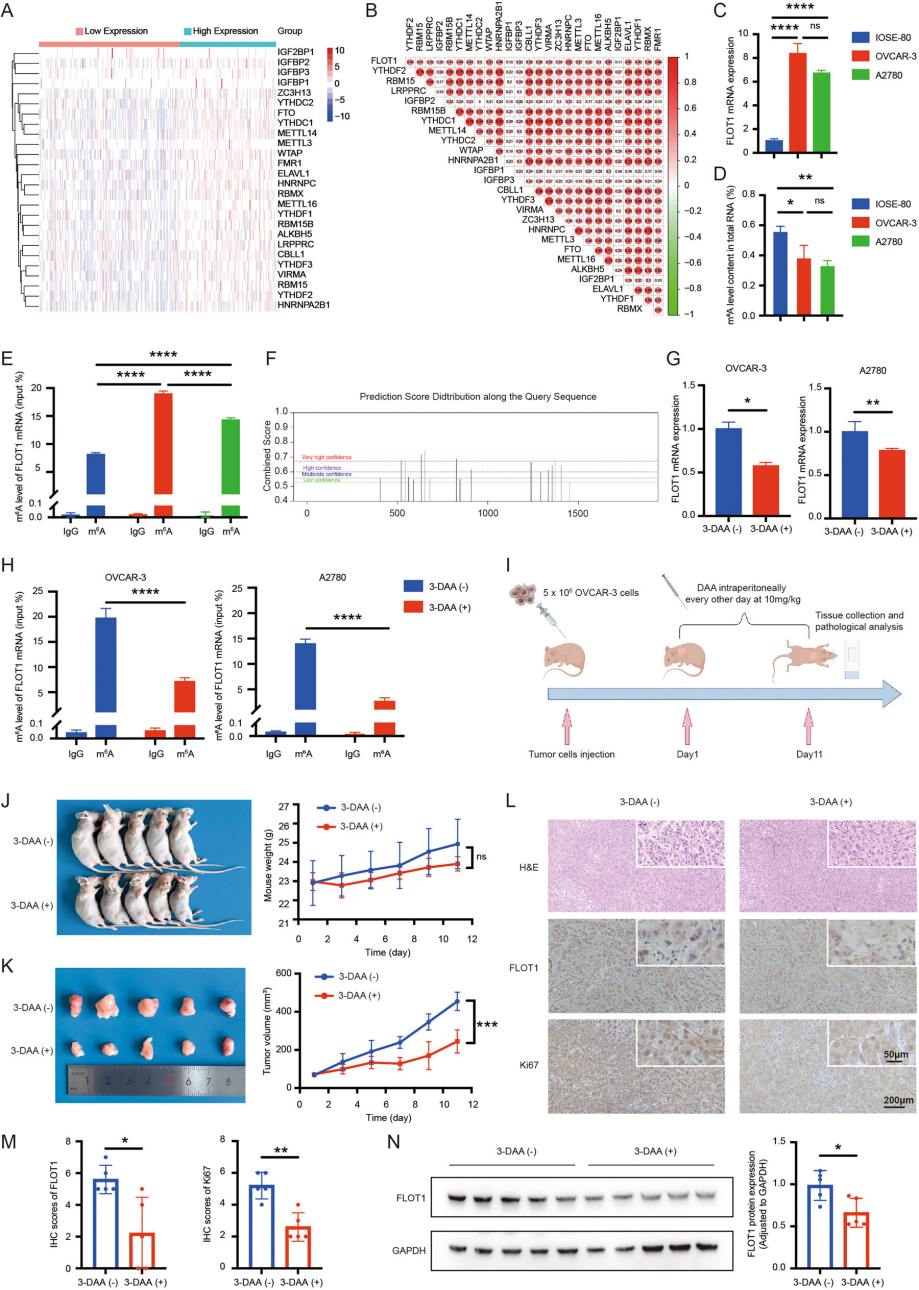
Effect of m^6^A modification on FLOT1 expression and tumor formation. (A) Heatmap showing unsupervised clustering for 26 genes of m^6^A RNA modification in OC patients (*n* = 376). Each column represents patients, and each row represents an m^6^A RNA modification regulator. (B) Heatmap of the correlation coefficient for the interaction between FLOT1 and the m^6^A RNA modification regulator. Red indicates a positive correlation, and green indicates a negative correlation. (C) Detection of FLOT1 mRNA expression in two human ovarian cancer cell lines (OVCAR‐3 and A2780) and a normal human ovarian surface epithelial cell line (IOSE‐80) by RT‐qPCR. Two‑way ANOVA followed by the Sidak test was used. The level of FLOT1 mRNA expression was higher in OVCAR‐3 and A2780 cells than in IOSE‐80 cells (*n* = 3). (D) The levels of total m^6^A methylated RNAs in OC cells were verified by colorimetrically quantified (*n* = 3). (E) Detection of the m^6^A modification level of FLOT1 mRNA in IOSE‐80, OVCAR‐3, and A2780. (F) Prediction of FLOT1 m^6^A modification sites by SRAMP. (G) Measurement of FLOT1 mRNA expression in OVCAR‐3 and A2780 cells detected by RT‐qPCR after 3‐deazaadenosine (3‐DAA) treatment. A Student *t*‐test was used (*n* = 3). (H) Detection of the m^6^A modification level of FLOT1 mRNA by MeRIP‑qPCR after 3‐DAA treatment. A Student *t*‐test was used (*n* = 3). (I) The workflow of ovarian cancer xenograft model construction and 3‐DAA treatment. (J) Xenograft tumor formation in nude mice. OVCAR‐3 cells were subcutaneously implanted in nude mice (*n* = 5/group). Animals were photographed on day 30. Measurement of mouse weight. (K) Photo of executed tumors. Live measurement of tumor volume. (L) Images of the H&E and immunohistochemistry staining of FLOT1 and Ki67. Original magnification x200; Scale bar, 200 µm. Partial magnification x600; Scale bar, 50 µm. (M) IHC staining score (ISS) of FLOT1 and Ki67 protein expression. (N) Evaluation of FLOT1 protein expression by Western blot and semi‐quantification of the blot in the 3‐DAA treated (+) and untreated (−) groups. The data are shown in the form of the mean ± SD. A Student *t*‐test was used. ns, not sigificance; *p < 0.05; **p < 0.01; ****p < 0.0001.

M^6^A modification plays a vital role in the regulation of gene expression via modulating the stability of substrate mRNAs. We first compared the expression levels of m^6^A regulatory genes between 376 OC samples from the TCGA database (https://portal.gdc.cancer.gov/projects/TCGA-OV). Using unsupervised cluster analysis, 26 genes of m^6^A RNA modification were identified (Figure 7A). Most of these genes showed higher expression in FLOT1‐overexpressed OC samples than in the low expression of FLOT1 OC samples, including FTO, YTHDC1, YTHDC2, METTL14, WTAP, FMR1, ELAVL1, RBMX, METTL16, YTHDF1, RBM15B, and ALKBH5. The correlation coefficients of these regulators were detected using the Pearson method. The results demonstrated that the top three genes related to FLOT1 were HNRNPC (R = 0.82), WTAP (R = 0.51), and FMR1(R = 0.49) and among the 26 regulatory genes, YTHDC1 and HNRNPA2B1 had the strongest correlation (R = 0.63) (Figure 7B). These data suggest that m^6^A regulatory factors, especially the writer WTAP and readers HNRNPC and FMR1 may affect FLOT1 expression. To further explore the specific molecular mechanism regulating FLOT1 expression in OC, we investigated whether a related mechanism was involved in the regulation of FLOT1 expression in OC. Compared with the normal human ovarian surface epithelial (IOSE‐80) cells, FLOT1 expression was higher in OC cells (OVCAR‑3 and A2780) at the mRNA levels detected by RT‑qPCR (Figure 7C). Although colorimetrically quantified m^6^A RNA methylation demonstrated that OC cells had a low level of total m^6^A‐methylated RNAs compared to normal ovarian epithelial cells (Figure 7D), the level of m^6^A‐methylated FLOT1 mRNA was higher in OVCAR‐3 and A2780 cells than in IOSE‐80 cells (Figure 7E). Furthermore, several candidate m^6^A sites of FLOT1 mRNA were predicted by the SRAMP database (http://www.cuilab.cn/sramp) (Figure 7F). The effect of a general methylation inhibitor 3‐DAA on FLOT1 mRNA expression was validated by RT‐qPCR and showed the suppression of FLOT1 mRNA by 3‐DAA (Figure 7G). Subsequently, the MeRIP‑qPCR assay using a specific pair of primers that covered 521‐651 sites demonstrated that 3‐DAA significantly reduced the m^6^A‐methylated FLOT1 mRNA level (Figure 7H). These data suggest that FLOT1 mRNA expression was affected by m^6^A modifications. To examine whether the regulation of FLOT1 mRNA expression levels by m^6^A methylation modification may influence the malignant behavior of tumors, a xenograft mouse model was generated (Figure 7I). OVCAR‐3 cells were subcutaneously injected into nude mice to validate the effect of 3‐DAA on tumor growth in vivo. After 3‐DAA injection, tumor formation was suppressed in mice (Figure 7J). The tumor volumes were decreased in 3‐DAA mice, indicating that the reduction of methylation levels suppressed tumor growth (Figure 7K). IHC staining analysis of the tumor tissues revealed that treatment with 3‐DAA inhibited tumor formation with the suppression of FLOT1 and the proliferation marker Ki67 protein expression (Figure 7L,M). The effect of 3‐DAA on FLOT1 expression was further evaluated by Western blot (Figure 7N). The semi‐quantification of the analysis revealed a significant reduction of FLOT1 expression in the 3‐DAA treated group compared to the untreated group.

## 4 Discussion

The current study demonstrated that FLOT1 is a novel biomarker, similar to CA125, for the detection of human OC. To our knowledge, this is the first report of FLOT1 as a serum biomarker of OC and can form a diagnostic panel in combination with CA125 to detect OC. Furthermore, FLOT1 has m^6^A methylation modification, which may be responsible for increasing the expression level of FLOT1 mRNA. Inhibiting the m^6^A modification by 3‐DAA decreased the level of FLOT1 mRNA and suppressed tumor formation.

Circulating biomarkers are regarded as reliable indicators in the diagnosis, monitoring, and prognosis of numerous cancers. Some serum biomarkers have already been applied in the clinical diagnosis of OC. CA125, also known as mucin 16 encoded by the *MUC16* gene, has been used for OC detection (Bast et al. 1981, Yin et al. 2002). However, there is a limitation as it lacks sensitivity and specificity, particularly in OC at early stages (Nossov et al. 2008). Our data showed the total detection rate of CA125 is 72.22% (70.00% at early stages of OC and 75.00% at late stages of OC), whereas the total detection rate of FLOT1 is 77.78% (70.00% at early stages of OC and 87.50% at late stages of OC). It has been shown that an increased blood CA125 is found in some patients with OC (Chudecka‐Glaz et al. 2009, Jacobs and Bast 1989). CA125 is elevated in 85% of serous, 65% of endometrioid, 40% of clear‐cell, 36% of undifferentiated, and only 12% of mucinous ovarian carcinomas (Hogdall et al. 2007). In addition, it also gives a false positive for inflammations, benign tumors, and other cancers, such as endometriosis (Wei et al. 2016), menstruation (Nossov et al. 2008), pregnancy (Sarandakou et al. 2007), liver cirrhosis (Chowdhury et al. 2014), breast cancer (Ertl et al. 2009), colorectal cancer (Huang et al. 2016), and pancreatic cancer (Liu et al. 2016).

Currently, human epididymis protein 4 (HE4), also known as WAP four‐disulfide core domain protein 2 encoded by the WFDC2 gene, is also reported as a tumor marker of OC (Hellström et al. 2003, Molina et al. 2011). However, the results of these studies are still controversial. Some studies report that the sensitivity and specificity of HE4 are not good enough compared with CA125 for OC prediction (Jia et al. 2016, Li et al. 2012, Moore et al. 2014), while others show that the sensitivity and specificity of HE4 are higher than CA125 (Ferraro et al. 2013, Wu et al. 2012), thus leading to two conclusions: the highest sensitivity (Abdel‐Azeez et al. 2010) and no benefit (Jacob et al. 2011) in the combination of HE4 and CA125 for detecting OC. Nevertheless, HE4 has not been used widely in the clinic and needs to be investigated further in the future.

It has been shown that FLOT1 plays an important role in many types of cancer, such as breast cancer (Koh et al. 2016), gastric cancer (Gao et al. 2015), nasopharyngeal carcinoma (Cao et al. 2016), clear‐cell renal cell carcinoma (Tomiyama et al. 2014), bladder transitional cell carcinoma (Guan et al. 2014), oral squamous cell carcinoma (Xiong et al. 2013), hepatocellular carcinoma (Zhang et al. 2013), tongue squamous cell cancer (Li et al. 2014b), non‐small cell lung cancer (Li et al. 2014a), and OC from our group (Li et al. 2018). We previously demonstrated that FLOT1 is overexpressed in OC cells. Knockdown of FLOT1 by FLOT1‐siRNA inhibits the proliferation of OC cells and arrests the cell cycle at the S phase, whereas suppression of FLOT1 increases cyclin E1 protein, indicating that FLOT1 plays an important role in ovarian tumor growth.

FLOT1 is recognized as a scaffolding protein linked with lipid raft microdomains not only in cancer cells but also presented in neuron projection terminus and distal axon as shown in the cellular component of our GO term enrichment analysis. FLOT1 is involved in signal transduction, cell adhesion, cytoskeletal remodeling, endocytosis, and signaling pathways (McClellan et al. 2024, Wang et al. 2023, Zhan et al. 2023). It has been shown that FLOT1 is an upstream regulator of the AKT/FOXO3a pathway, affecting cell proliferation in breast cancer (Lin et al. 2011) by accelerating the cell cycle, inhibiting apoptosis, and initiating EMT through the AKT, TGF‐β, and NF‐κB pathways (Cao et al. 2016, Song et al. 2012, Wei et al. 2022), which is consistent with our KEGG pathway analysis. Although small molecule kinase inhibitors like those targeting PI3K/AKT show potential in cancer treatment, single‐target inhibitors often lead to drug resistance due to the interaction of tumor growth signals. Identifying common upstream regulators of various survival signals as potential intervention targets in cancer therapy may enhance anticancer efficacy and stability (He et al. 2021). Given that lipid rafts function as platforms for signal transduction, targeting proteins associated with lipid rafts could result in greater antitumor efficacy by simultaneously deactivating multiple pro‐tumor signaling pathways (Gauthier‐Rouvière et al. 2020).

It was reported that FLOT1 could be a potential biomarker candidate for invasive breast, gastric, and lung cancers (Zhang et al. 2012) but no such report about FLOT1 in OC. Screening analysis of the public databases showed a high FLOT1 expression in OC tissues and cells compared with normal ovary controls. Furthermore, the level of serum FLOT1 was significantly difference between ovarian benign and malignant tumors. Our ROC analyzes support the fact that FLOT1 can distinguish OC patients from healthy controls with slightly higher sensitivity and specificity than CA125.

There was no significant correlation between FLOT1 and CA125 in healthy controls and patients with OC, indicating that FLOT1 is an independent biomarker. In the view of diagnosis, FLOT1 in combination with CA125 can significantly enhance the detection rate of OC. A panel of FLOT1 and CA125 can be used not only for increasing the sensitivity but also for increasing the accuracy, providing an opportunity to further assess the diagnosis of OC. To confirm that FLOT1 is a serum biomarker of OC, a xenograft mouse model was used. Serum FLOT1 of animals was significantly higher in the subcutaneous injection mice than in control mice. We also found that serum FLOT1 was significantly increased in patients with OC compared to healthy controls. Thus, we speculate that FLOT1 is a valuable biomarker of OC detection and can assist clinicians in detecting OC, indicating that FLOT1 is a potential candidate biomarker. Some studies showed that FLOT1 was negatively associated with cancer survival, including hepatocellular carcinoma (Zhang et al. 2013), tongue squamous cell cancer (Li et al. 2014b), and non‐small cell lung cancer (Li et al. 2014a). These cancer patients with higher FLOT1 expression had shorter OS time, whereas those with lower FLOT1 expression had longer survival time. However, no data on FLOT1 in OC survival has been reported previously.

M^6^A modification is widely implicated in various biological processes. Nevertheless, the role of m^6^A modification in human diseases, especially in OC, was previously not well studied. In this study, RT‐qPCR assay verified that methylation inhibitors significantly decreased the mRNA expression of FLOT1 in OC cells. The MeRIP‐qPCR assay demonstrated a significant decrease in the m^6^A methylated FLOT1 mRNA level after a methylation inhibitor treatment. 3‐DAA has been widely used as a broad‐spectrum methylation inhibitor because it damages the s‐adenosylmethionine cycle, which offers a methyl donor (Chiang et al. 1977). Our findings indicate that m^6^A modification plays a pivotal role in the oncogenic function of FLOT1 in OC.

There are some limitations in the current study. First of all, the sample size of patients is relatively small and the OC subtype is not classified. More adequate clinical features will need to be collected and the number of cases will need to be increased in the future study. Second, the identification of the mechanism of m^6^A modification is required further. Third, a clinical trial may be conducted.

## 5 Conclusion

The present study identified FLOT1 as a novel serum biomarker of OC. A combination with CA125 consisting of a diagnostic panel of FLOT1 may be used as an optimal tool to improve the accuracy of OC detection. Methylation inhibitors decreased FLOT1 mRNA expression in OC cells. Targeting m^6^A methylated FLOT1 mRNA may provide a novel therapeutic strategy for the treatment of patients with OC. It may be important to monitor serum FLOT1 levels pretreatment and the changing pattern of FLOT1 posttreatment for judging prognosis and diagnosing recurrences of OC.

## Author Contributions

Bin Guan: Data curation, Software, Formal analysis, Validation, Investigation, Visualization, Writing‐original draft. Qi Lu, Junyu Chen, Jingyi Fang: Formal analysis, Validation, Investigation, Visualization. Zhenyu Liu, Wei Li: Methodology. Lingyun Zhang, G.X: Conceptualization, Resources, Data curation, Supervision, Funding acquisition, Project administration, Writing‐review, and editing. All authors read and approved the final manuscript.

## Ethics Statement

This study was approved by the Ethics Committee of Jinshan Hospitals of Fudan University (approval no. JYLL‐2013‐019‐01) and the Ethics Committee of Zhongshan Hospital of Fudan University (approval no. B2023‐023 R). Written informed consent was obtained from all patients or their families in this study. The animal study was approved by the Laboratory Animal Welfare and Ethics Committee of the Shanghai Public Health Clinical Center (Approval No. 2020‐A027‐01).

## Conflicts of Interest

Z. Liu and W. Li are the employees, cofounders, and leaders of Shanghai Yizhi Medical Technology Co. Ltd, Shanghai, China. The other authors declare no conflicts of interest.

## Data Availability

The data that support the findings of this study are available from the corresponding author upon reasonable request.
